# Myelin Oligodendrocyte Glycoprotein Antibody-Associated Disease Complicated by Pachymeningitis: A Case Report

**DOI:** 10.7759/cureus.64868

**Published:** 2024-07-18

**Authors:** Sanjay M Khaladkar, Prajakta P KirdatPatil, Aryaman Dhande, Neeha A Jhala

**Affiliations:** 1 Radiodiagnosis, Dr. D. Y. Patil Medical College, Hospital and Research Centre, Dr. D. Y. Patil Vidyapeeth, Pune, IND

**Keywords:** demyelination, dural enhancement, mog (myelin oligodendrocyte glycoprotein), mogad(myelin oligodendrocyte glycoprotein antibody associated disease), pachymeningitis

## Abstract

Myelin oligodendrocyte glycoprotein antibody-associated disease (MOGAD) is a rare autoimmune disorder that primarily affects the central nervous system (CNS). We present a unique case of MOGAD complicated by pachymeningitis, which is characterized by inflammation of the dura mater. The clinical presentation included vertigo, nausea, and vomiting. A diagnostic workup confirmed MOGAD complicated by pachymeningitis. This case underscores the diverse clinical manifestations of MOGAD and highlights the challenges in diagnosis and management, particularly when complicated by rare manifestations like pachymeningitis.

## Introduction

Myelin oligodendrocyte glycoprotein antibody-associated disease (MOGAD) is recognized as a distinct entity among autoimmune disorders affecting the central nervous system (CNS). It mainly attacks the myelin sheath and oligodendrocytes, which can cause several neurological problems including optic neuritis, transverse myelitis, and acute disseminated encephalomyelitis (ADEM) [1]. MOGAD is characterized by the presence of antibodies against myelin oligodendrocyte glycoprotein (MOG), distinct from those seen in multiple sclerosis (MS).

Men are more affected than women. Its clinical presentation varies with age; younger children usually present with ADEM, while older children (>9 years) and adults typically present with optic neuritis and longitudinally extensive transverse myelitis [2]. The prevalence is higher in children due to their higher presence of MOG antibodies [3]. Hypertrophic pachymeningitis usually presents with headaches, cranial nerve dysfunction, and rarely, seizures. Myelin oligodendrocyte glycoprotein is exclusively found in the CNS [2]. Pachymeningitis, or inflammation of the dura mater, is a rare complication of MOGAD that has been scarcely reported in the literature. Here, we present a case of MOGAD complicated by pachymeningitis, detailing the clinical course, diagnostic challenges, therapeutic interventions, and outcomes. By documenting such cases, we aim to enhance our understanding of the spectrum of MOGAD presentations and contribute to the management strategies for this rare autoimmune disorder.

## Case presentation

A 35-year-old male patient presented with vertigo-related nausea and vomiting persisting for three days, accompanied by horizontal gaze-evoked nystagmus to the left side, headache, and newly diagnosed hypertension. The Romberg test was positive on clinical examination with no cerebellar signs, and the Dix-Hallpike maneuver was positive. There was no significant past or family history. Vestibular neuritis was suspected based on physical and clinical examination.

Bilateral pure tone audiometry (PTA) and two-dimensional (2D) echocardiography tests were normal. Laboratory findings showed normal infectious screening of serum and CSF. The CSF examination revealed occasional red blood cells, no cobweb. MOG immunoglobulin G (IgG) antibodies in CSF were positive, while neuromyelitis optica (NMO) IgG antibodies were negative by cell-based assay (CBA). It also showed mildly elevated protein levels and normal glucose levels (Table 1).

**Table 1:** CSF analysis.

Chemical examination	Result	Reference value	Unit
Protein	50.5	15-45	mg/dl
Glucose	68	40-70	mg/dl

**Figure 1:**
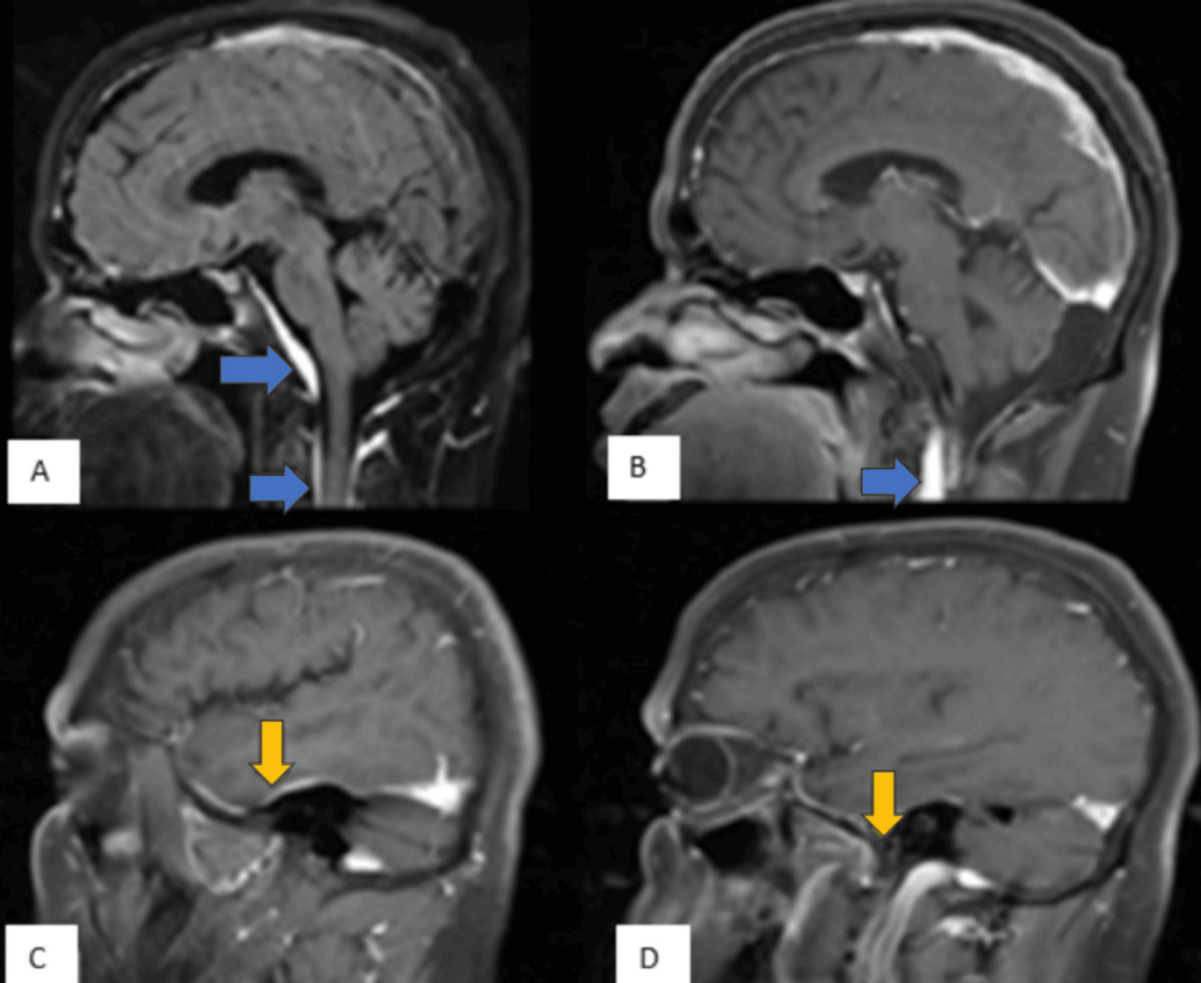
(A) Sagittal FLAIR post-contrast and (B-D) sagittal T1FS post-contrast images show dense pachymeningeal enhancement. Overlying the clivus and in the upper spinal canal, just anterior and posterior to the upper cervical cord, are marked by blue arrows (A, B). Yellow arrows (C, D) indicate the location of both temporal lobes. FLAIR: Fluid-attenuated inversion recovery; T1FS: T1-weighted fat saturated.

MRI showed normal parenchyma; however, there was dense pachymeningeal enhancement over the clivus and in the upper spinal canal, both in front of and behind the upper cervical cord (Figure 1). This was also seen in both temporal lobes. The bilateral VII and VIII nerve complexes and internal auditory canals were normal. The rest of the cranial nerves were also normal.

The patient underwent a five-day course of methylprednisolone 1 gm in 300 ml of normal saline once daily (OD), followed by prednisolone 50 mg OD for five days, tapered to 40 mg for five days, and further tapered to 10 mg. The patient also received vitamin D3 + calcium (500 mg OD), aspirin (150 mg OD), esomeprazole (40 mg OD), and cilnidipine + telmisartan (10/40 mg OD). After one week, he showed improvement in his symptoms and received instructions for follow-up in the outpatient department.

## Discussion

MOGAD is an inflammatory disorder in the CNS. It involves immune-mediated demyelination that affects various parts of the CNS, including the optic nerves, brain, and spinal cord. As a result, MRI scans often show findings such as optic neuritis, ADEM, brainstem encephalitis, and myelitis [4]. MOGAD is distinct from MS and NMO spectrum disorders [5]. MOGAD represents a novel entity associated with antibodies. Hypertrophic pachymeningitis is a rare presentation. The pathognomonic MRI findings of hypertrophic pachymeningitis include abnormal dural enhancement [5]. MOG is located on the myelin surface [6]. CSF exhibits pleocytosis, with more than 50 WBCs; oligoclonal bands are typically not formed. A CBA can detect MOG antibodies in serum, and MOG antibodies in CSF are typically low, making them difficult to measure [2].

In MOGAD, meningitis/meningoencephalitis with leptomeningeal enhancement was previously described [5]. However, Ueno et al. (2019) reported dura mater involvement [7]. Hypertrophic pachymeningitis is characterized by dural thickening and contrast enhancement. The MR venography is normal [8].

Hypertrophic pachymeningitis caused by MOGAD is clinically indistinguishable from other causes of hypertrophic pachymeningitis (Table 2) and occurs due to mechanical compression of vessels and nerves, leading to functional deficits [9, 10]. The condition typically presents as a headache. The second most common manifestation is cranial nerve involvement. The optic nerve is most commonly involved, followed by the III, IV, V, VI, and VIII nerves, in that order [11]. Other clinical features include hearing loss, seizures, sensory impairment, ataxia, and dyskinesia. The incidence is 0.949 per 100,000 [12].

**Table 2:** Causes of pachymeningitis. Source: [9,10].

Etiology	Causes
Inflammatory conditions	IgG4-related disease, Behçet's disease, rheumatoid arthritis, Sjögren’s syndrome, temporal arteritis, sarcoidosis, granulomatosis with polyangiitis
Infectious	Tuberculosis, cysticercosis, fungal infection, syphilis, Lyme disease
Malignant	Dural carcinomatosis, lymphoma, metastatic disease, meningioma
Intracranial hypotension	Spontaneous or after spinal fluid drainage

The early stages often result in missed and misdiagnosed cases due to the non-specific and mild symptoms. Delayed diagnosis and treatment result in multisite involvement of the nervous system, with chances of recurrence and a poor prognosis [11].

The exact pathogenesis of MOGAD-hypertrophic pachymeningitis is unknown, as the dura mater lacks oligodendrocytes. However, it is likely that MOG antibodies interact with heterotopic neurological tissue present in the dura mater [5].

Treatment includes steroids and immunosuppressive drugs such as methotrexate and rituximab. Usually, there is no coexisting systemic immunity [4]. In the acute phase, we recommend high-dose IV glucocorticoids for 3-5 days. If IV steroids do not lead to improvement, we use IV immunoglobulin (IVIG) therapy. As the disease is monophasic, long-term management remains controversial. Due to the high risk of flare-ups within two months of discontinuation, steroids should be tapered slowly, ensuring that prednisolone does not exceed 10 mg per day [2].

## Conclusions

The case illustrates the complexity and variability of symptoms associated with MOGAD, especially when complicated by pachymeningitis. This rare autoimmune disorder highlights the importance of a comprehensive diagnostic approach that includes serological testing for MOG antibodies, neuroimaging, and CSF analysis. Managing such cases requires a multidisciplinary approach tailored to address both the autoimmune pathophysiology and the specific manifestations of pachymeningitis.
